# Preparation and Hepatoprotective Activities of Peptides Derived from Mussels (*Mytilus edulis*) and Clams (*Ruditapes philippinarum*)

**DOI:** 10.3390/md20110719

**Published:** 2022-11-16

**Authors:** Qian Wang, Fu-Jun Liu, Xin-Miao Wang, Guan-Hua Zhao, Dong Cai, Jing-Han Yu, Fa-Wen Yin, Da-Yong Zhou

**Affiliations:** 1 School of Food Science and Technology, Dalian Polytechnic University, Dalian 116034, China; 2 Liao Fishing Group Limited Company, Dalian 116000, China; 3 National Engineering Research Center of Seafood, Dalian Polytechnic University, Dalian 116034, China; 4 Collaborative Innovation Center of Seafood Deep Processing, Dalian 116034, China

**Keywords:** *Mytilus edulis*, *Ruditapes philippinarum*, hepatoprotective peptide, H_2_O_2_-induced oxidative damage, mice, acute liver injury

## Abstract

Low molecular weight (<5 kDa) peptides from mussels (*Mytilus edulis*) (MPs) and the peptides from clams (*Ruditapes philippinarum*) (CPs) were prepared through enzymatic hydrolysis by proteases (dispase, pepsin, trypsin, alcalase and papain). Both the MPs and the CPs showed excellent in vitro scavenging ability of free radicals including OH, DPPH and ABTS in the concentration range of 0.625–10.000 mg/mL. By contrast, the MPs hydrolyzed by alcalase (MPs-A) and the CPs hydrolyzed by dispase (CPs-D) had the highest antioxidant activities. Furthermore, MPs-A and CPs-D exhibited protective capabilities against oxidative damage induced by H_2_O_2_ in HepG2 cells in the concentration range of 25–800 μg/mL. Meanwhile, compared with the corresponding indicators of the negative control (alcohol-fed) mice, lower contents of hepatic MDA and serums ALT and AST, as well as higher activities of hepatic SOD and GSH-PX were observed in experiment mice treated with MPs-A and CPs-D. The present results clearly indicated that *Mytilus edulis* and *Ruditapes philippinarum* are good sources of hepatoprotective peptides.

## 1. Introduction

Mussels (*Mytilus edulis*) and clams (*Ruditapes philippinarum*) are two low-cost economical marine bivalve shellfish. According to statistics from the Food and Agriculture Organization of the United Nations (FAO) [1], the annual global production of *Mytilus edulis* and *Ruditapes philippinarum* reached 1108.3 and 4266.2 million tons in 2020. Currently, most *Mytilus edulis* and *Ruditapes philippinarum* are sold as either fresh or dried products. Hence, there is an urgent need to develop high-value-added products, and further increase their economic value.

Seafood, such as bivalve shellfish, is a delicacy consumed all over the world. Meanwhile, they may also serve as a rich source of health-beneficial ingredients including proteins, peptides, essential amino acids, omega-3 long-chain polyunsaturated fatty acids, minerals and vitamins [2,3,4]. Many studies have demonstrated that the striped mussel (*Mytilus edulis*) contains a high percentage of protein (60–65% of dry weight) and essential amino acids (35–45% of total amino acids) [5,6]. The striped clam (*Ruditapes philippinarum*) also contains a high percentage of protein (65–75% of dry weight) and essential amino acids (35–45% of total amino acids) [7,8].

Among the above-mentioned bioactive ingredients, proteins have shown the strongest potential for the commercial exploitation of functional foods or dietary supplements up until now. To date, the studies of bioactive peptides such as antioxidant peptides, ACE inhibitory peptides, anticancer peptides and anticoagulant peptides from *Mytilus Edulis* and *Ruditapes philippinarum* hydrolyzed by various proteases have been widely reported [9,10,11]. For example, Wang et al. reported that an antioxidant peptide was successfully isolated from the hydrolysate of blue mussels (*Mytilus edulis*) by neutrase, which displayed good radical scavenging activity. In the model system of linoleic acid, the peptide had a significant effect on anti-lipid peroxidation [9]. Qiao et al. reported that an anticoagulant peptide was produced from the hydrolysate of *Mytilus edulis* by trypsin. The excellent anticoagulant activity was probably attributed to its high-affinity interaction with thrombin [10]. As for clams (*Ruditapes philippinarum*), Song et al. reported that peptides (RBPs) with higher ACE inhibition function were produced by fermentation of *Ruditapes philippinarum* inoculated with *Bacillus natto* [11]. Furthermore, by decreasing the proportion of *Firmicutes* and *Bacteroidetes* and increasing the relative abundance of certain genera, such as *Ruminococcaceae_UCG-014*, RBPs could improve the intestinal microbiota. In addition, Kim et al. reported that a novel anticancer peptide extracted from clams (*Ruditapes philippinarum*) by chymotrypsin could effectively induce apoptosis in prostate, breast and lung cancer cells except normal hepatocytes [12]. However, to the best of our knowledge, the preparation of hepatoprotective peptides from *Mytilus edulis* and *Ruditapes philippinarum* has not been reported.

It has been widely reported that acute ethanol administration could increase oxidative stress, decrease motor coordination, decrease the respiratory rate and impair protein metabolism [13]. Especially, excessive alcohol intake can cause liver injury, which may aggravate gradually and possibly lead to alcoholic liver disease (ALD). In general, ALD is generally characterized by liver injury and numerous inflammatory cytokines infiltration reactions, which will lead to more serious liver disease or pathological evolution [14]. With the aggravation of the disease, ALD may further lead to serious liver disease-related morbidity and mortality [15]. Therefore, nutritional intervention in the early stage of acute liver injury is of crucial significance for body health. So far, researchers have successfully isolated and prepared hepatoprotective peptides from red shrimp, crucian carp, freshwater clam and other raw materials, which could effectively inhibit acute induced liver injury [16,17,18,19]. For example, Jiang et al. reported that based on inhibiting the NF-κB signal responses and reducing the expression of the inflammatory factors (IL-1β, IL-6, IFN-γ and TNF-α), the low molecular weight peptides (SCHPs-F1) from red shrimp (*Solenocera crassicornis*) head significantly ameliorate the cyclophosphamide-induced hepatotoxicity [19]. Shi et al. reported that a peptide with the sequence Gly-Leu-Hyp-Gly-Glu-Arg (GLpGER) extracted from the swim bladder hydrolysate of crucian carp (*Carassius auratus*) could alleviate acute alcoholic liver injury, it could restore liver alcohol dehydrogenase (ADH) activity, maintain the normal morphology of hepatocytes and decrease the serum alanine aminotransferase and aspartate aminotransferase levels [16]. Je et al. reported that serum markers of liver injury in rats, including alanine aminotransferase, aspartate aminotransferase, alkaline phosphatase and lactate dehydrogenase, were significantly (*p* < 0.05) increased after alcohol administration for 4 weeks. Nevertheless, pepsin-hydrolyzed bioactive peptides derived from the pectoral fin of salmon (*Oncorhynchus*) resulted in a significant (*p* < 0.05) reduction in the above indicators. The results indicated that such peptides could provide a hepatoprotective effect on the liver damaged by alcohol, which was also confirmed by the evaluation of liver histopathology [20].

Currently, in vitro models of cell injury and animal models of acute liver injury are widely used in the related studies of active peptides with hepatoprotective effects. Since hydrogen peroxide (H_2_O_2_) is readily converted to hydroxyl radicals, one of the most destructive free radicals, it is an important cause of intracellular oxidative damage [21]. Moreover, the hydroxyl radical is generated from nearly all sources of oxidative stress and can diffuse freely in and out of the cells and the tissues [22]. Therefore, H_2_O_2_ can trigger apoptosis in hepatocytes and many researchers usually choose it to establish a human hepatocellular carcinomas (HepG2) cells injury model. In addition, experimental animal models of alcoholic liver injury, particularly rodents, have been widely used to mimic human alcoholic liver injury because they are suitable for most experiments, and have the advantages of being economical and shortening experimental periods compared with other animal models, although rodents (mainly mice and rats) cannot exhibit the full spectrum of disease in human alcoholic liver injury as primates do [14,23,24]. In consequence, many researchers are more likely to choose mice as experimental animals to establish an animal model of alcohol-induced acute liver injury. In general, the simultaneous use of the above two models could more effectively evaluate the hepatoprotective activity or potential mechanism of functional ingredients such as bioactive peptides, which will further facilitate the preparation and development of hepatoprotective peptides.

Given this, the in vitro and the in vivo hepatoprotective effects of peptides from *Mytilus edulis* and *Ruditapes philippinarum* were evaluated in this study. The obtained experimental data will provide a theoretical basis for the utilization of hepatoprotective peptides from *Mytilus edulis* and *Ruditapes philippinarum* as novel sources of ingredients for value-added nutritious foods. It will effectively improve the economic value of low-cost economical marine bivalve shellfish.

## 2. Results

### 2.1. Antioxidant Activities of Peptides from Mussels (Mytilus edulis) and Clams (Ruditapes philippinarum) Hydrolyzed by Five Proteases

Generally, hydroxyl radical (OH) radicals can induce oxidative modification in virtually all types of macromolecules including proteins and lipids resulting in cellular damage and cell death [25,26]; the 2,2-diphenyl-1-picrylhydrazyl (DPPH) radical scavenging assay is based on the principle of single-electron transport (SET), which has the advantages of easy operation, rapidness, automation, reproducibility and usability at ambient temperature [27,28,29]; 2,2′-azino-bis (3-ethylbenzothiazoline-6-sulfonic acid) (ABTS) produces a metastable cation when subjected to oxidation by H_2_O_2_ or ferry myoglobin [30]. 

**Figure 1:**
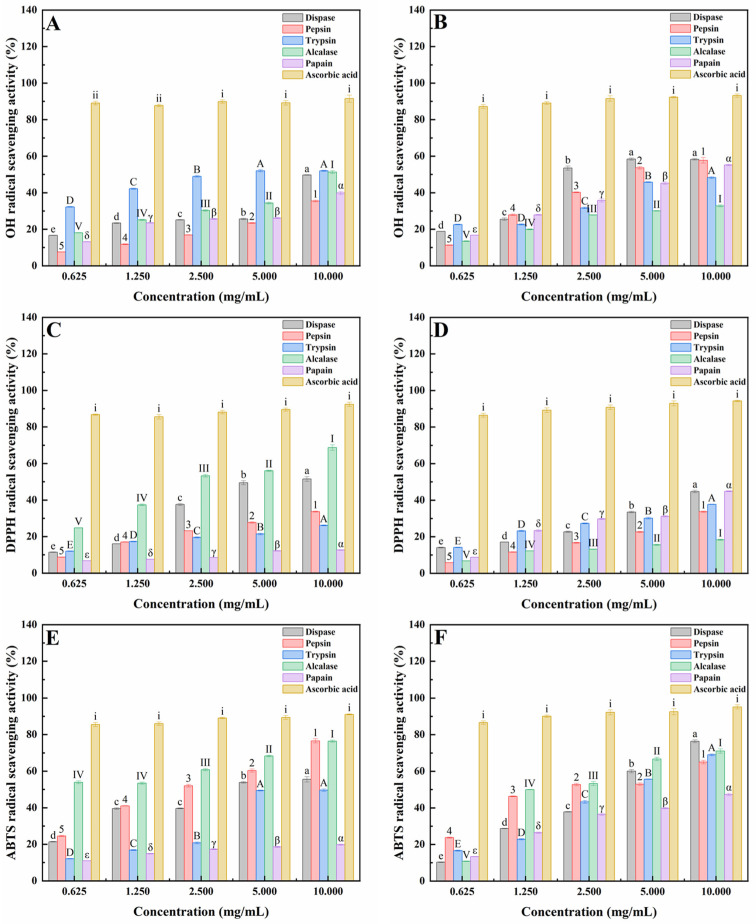
OH, DPPH and ABTS radicals scavenging activities of peptides from mussels (*Mytilus edulis*) (MPs; **A**,**C**,**E**) and clams (*Ruditapes philippinarum*) (CPs; **B**,**D**,**F**) hydrolyzed by five proteases. Data are expressed as the mean ± standard error, n = 8. Values of different groups with different lower-case letters (a–e), numbers (1–5), upper-case letters (A–E), upper-case Roman numerals (Ⅰ–Ⅴ) and Greek letters (α–ε) are significantly different at *p* < 0.05.

As shown in Figure 1, CPs from dispase and pepsin had the strongest free radical scavenging activity against OH. By contrast, CPs from dispase and papain had the strongest free radical scavenging activity against DPPH, and CPs from dispase and alcalase had the strongest free radical scavenging activity against ABTS.

The above results of OH, DPPH and ABTS radical scavenging experiments clearly indicated that MPs hydrolyzed by alcalase and CPs hydrolyzed by dispase had the highest antioxidant activities. Many studies have reported that antioxidant activity correlated with hepatoprotective activity [31,32]. Therefore, MPs hydrolyzed by alcalase (MPs-A) and CPs hydrolyzed by dispase (CPs-D) were chosen to evaluate the cytoprotective effects on HepG2 cells damaged by H_2_O_2_ oxidation.

### 2.2. Hepatoprotective Effect of MPs-A and CPs-D on H_2_O_2_-Induced Oxidative Damage in HepG2 Cells

#### 2.2.1. Establishment of a HepG2 Cell Model for In Vitro Hepatoprotective Activity Studies

**Figure 2:**
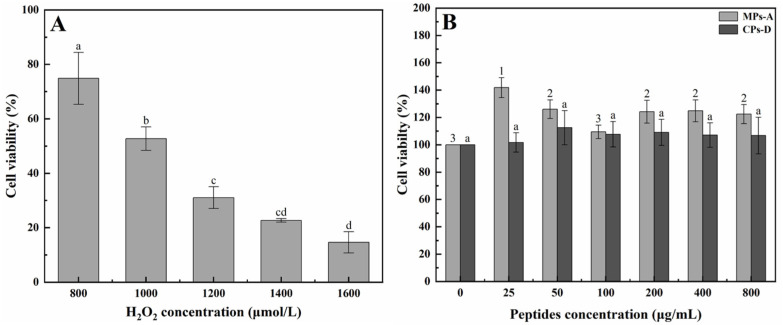
Cell viability of HepG2 cells treated with H_2_O_2_ (**A**), peptides from mussels (*Mytilus edulis*) hydrolyzed by alcalase (MPs-A) and clams (*Ruditapes philippinarum*) hydrolyzed by dispase (CPs-D) (**B**). Data are expressed as the mean ± standard error, *n* = 8. Values of different groups with different lower-case letters (a–d) and numbers (1–3) are significantly different at *p* < 0.05.

For the purpose of appraising the in vitro hepatoprotective activity of MPs and CPs, oxidative damage in HepG2 cells was induced by hydrogen peroxide (H_2_O_2_) treatment. H_2_O_2_, as a commonly used oxidant, has been used to induce oxidative stress leading to cell death in a variety of experimental models such as cell models [33]. In this study, cell viability was determined by the methyl thiazolyl tetrazolium (MTT) assay, which was used to evaluate the degree of cell death. As shown in Figure 2A, the addition of H_2_O_2_ to the cell culture medium caused cell death. H_2_O_2_ with concentrations ranging from 800 μmol/L to 1600 μmol/L induced a decrease in cell viability in a concentration-dependent manner. Especially, under the H_2_O_2_ concentration of 1000 μmol/L, appropriate cell viability of 50% was obtained. Therefore, 1000 μmol/L of H_2_O_2_ was chosen to induce oxidative damage in HepG2 cells in the following experiments. In addition, HepG2 cells were exposed to different concentrations of MPs-A and CPs-D in order to evaluate whether the peptides could damage the cells. As shown in Figure 2B, it was obvious that MPs-A and CPs-D did not cause any apparent cytotoxic effects on HepG2 when the concentrations ranged from 25 μg/mL to 800 μg/mL. Therefore, this concentration range of MPs-A and CPs-D would be used in subsequent experiments.

#### 2.2.2. Protective Effects of MPs-A and CPs-D on H_2_O_2_-Induced Oxidative Damage in HepG2 Cells

**Figure 3:**
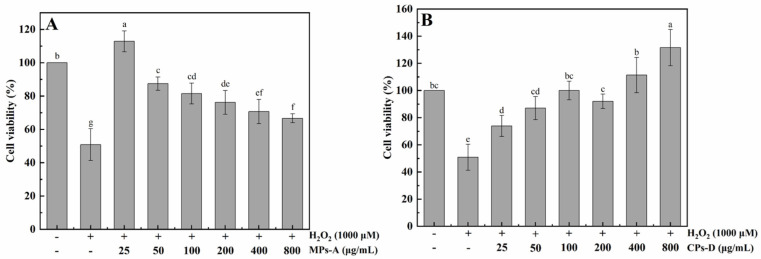
Protective effects of peptides from mussels (*Mytilus edulis*) hydrolyzed by alcalase (MPs-A) (**A**) and clams (*Ruditapes philippinarum*) hydrolyzed by dispase (CPs-D) (**B**) on H_2_O_2_-induced oxidative damage in HepG2 cells. Data are expressed as the mean ± standard error, n = 8. Values of different groups with different letters are significantly different at *p* < 0.05. −, the compound (H_2_O_2_, MPs-A or CPs-D) is not added in the liquid medium; +, H_2_O_2_ is added in the liquid medium.

The cytoprotection against H_2_O_2_-induced oxidative damage exerted by MPs-A and CPs-D is evaluated in this section. As shown in Figure 3, cell death induced by H_2_O_2_ was effectively inhibited by the addition of MPs-A and CPs-D. Both types of peptides showed excellent in vitro hepatoprotective activity in the concentration range of 25–800 μg/mL. The different tendencies between MPs-A and CPs-D were closely related to the molecular weight, the amino acid composition and the arrangement of the peptide chains, etc. By contrast, 25 μg/mL of MPs-A and 800 μg/mL CPs-D exhibited the strongest hepatoprotective activity. Compared with in vitro cell experiments, the in vivo biological evaluation can give more accurate information. Therefore, further animal experimentation is required to comprehensively evaluate the in vivo hepatoprotective activity of MPs-A and CPs-D.

### 2.3. Hepatoprotective Effects of MPs-A and CPs-D on Acute Alcohol-Induced Liver Injury in Mice

#### 2.3.1. Effects of MPs-A and CPs-D on Body Weight Gain, Liver Index and Serum Indexes

As shown in Table 1, body weight gain, liver index and serum indexes were measured to appraise the protective effect of MPs-A and CPs-D on alcohol-damaged mice. It was obvious that the liver index and the level of serum indexes including alanine transaminase (ALT), aspartate transaminase (AST), total cholesterol (TC) and triglyceride (TG) induced by alcohol (13 mL/kg BW) in alcohol control (AC) group were significantly higher than those in the water control (WC) group. Whereas the body weight gain in the AC group was lower than those in the WC group. The treatment of MPs-A and CPs-D significantly inhibited the alcohol-induced increase in ALT, AST, TC and TG in serum, and also increased the body weight gain. Based on the values of the above-mentioned indicators, the high-dose MPs-A group (MH, 600 mg/kg BW MPs) was found to exhibit excellent hepatoprotective effects in the groups treated with different doses of MPs-A. Similarly, the high-dose CPs-D group (CH, 600 mg/kg BW CPs) was found to exhibit excellent hepatoprotective effects in the groups treated with different doses of CPs-D.

#### 2.3.2. Effects of MPs-A and CPs-D on Hepatic MDA, GSH-PX and SOD

**Figure 4:**
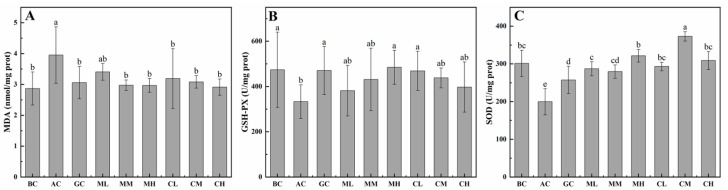
Effects of peptides from mussels (*Mytilus edulis*) hydrolyzed by alcalase (MPs-A) and clams (*Ruditapes philippinarum*) hydrolyzed by dispase (CPs-D) on alcohol-induced changes in hepatic MDA content (**A**), GSH-PX activity (**B**) and SOD activity (**C**). WC (water control), isometric distilled water; AC (alcohol control), 13 mL/kg body weight (BW) alcohol; GC (GSH control), 150 mL/kg BW GSH + 13 mL kg^−1^ BW alcohol; ML (low-dose MPs-A), 150 mL/kg BW MPs-A + 13 mL/kg BW alcohol; MM (medium-dose MPs-A), 300 mL/kg BW MPs-A + 13 mL/kg BW alcohol; MH (high-dose MPs-A), 600 mL/kg BW MPs-A + 13 mL/kg BW alcohol; CL (low-dose CPs-D), 150 mL/kg BW CPs-D + 13 mL/kg BW alcohol; CM (medium-dose CPs-D), 300 mL/kg BW CPs-D + 13 mL/kg BW alcohol; CH (high-dose CPs-D), 600 mL/kg BW CPs-D + 13 mL/kg BW alcohol. Data are expressed as the mean ± standard error, *n* = 8. Values of different groups with different letters are significantly different at *p* < 0.05.

As shown in Figure 4, malondialdehyde (MDA) content, glutathione peroxidase (GSH-PX) and superoxide dismutase (SOD) activities were detected to evaluate the protective effect of MPs-A and CPs-D on alcohol-induced injury in the liver. Obviously, the content of MDA in the alcohol control (AC) group was higher than that in the water control (WC) group. The treatment of MPs-A and CPs-D significantly inhibited the alcohol-induced increase in hepatic MDA, and also increased the activities of hepatic GSH-PX and SOD. Based on the values of the above-mentioned indicators, the high-dose MPs-A group (MH, 600 mg/kg BW MPs) was found to exhibit excellent hepatoprotective effect in the groups treated with different doses of MPs-A. Similarly, the high-dose CPs-D group (CH, 600 mg/kg BW CPs) was found to exhibit excellent hepatoprotective effects in the groups treated with different doses of CPs-D.

#### 2.3.3. Liver Histological Analysis

**Figure 5:**
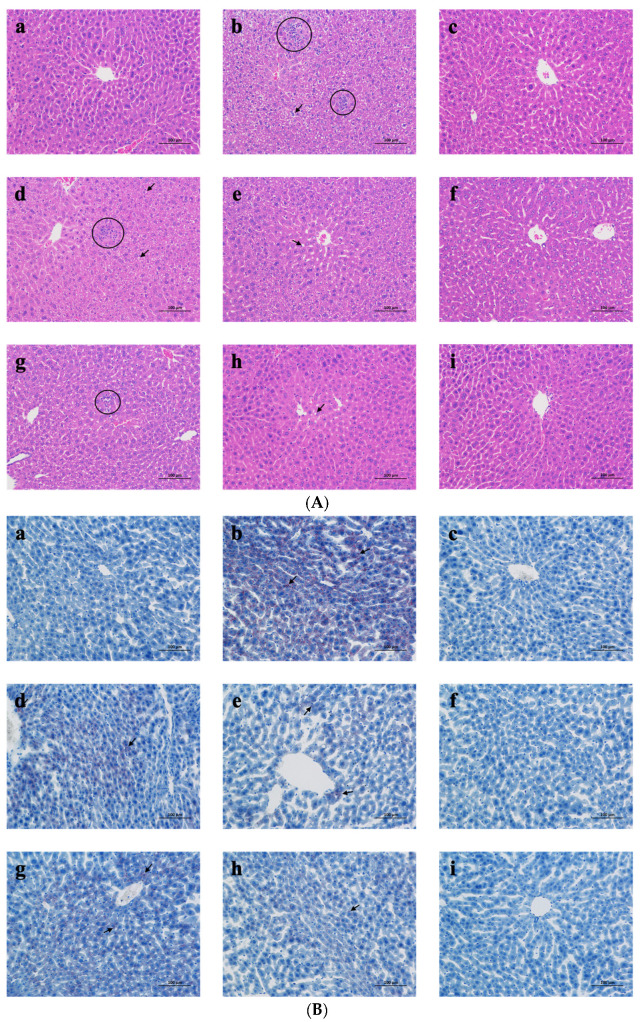
Effects of peptides from mussels (*Mytilus edulis*) hydrolyzed by alcalase (MPs-A) and clams (*Ruditapes philippinarum*) hydrolyzed by dispase (CPs-D) on alcohol-induced changes in histopathological sections (scale bar: 100 μm) stained with hematoxylin and eosin (H&E) (**A**) and Oil Red O (ORO) (**B**). (**a**), WC (water control), isometric distilled water; (**b**), AC (alcohol control), 13 mL/kg body weight (BW) alcohol; (**c**), GC (GSH control), 150 mL/kg BW GSH + 13 mL/kg BW alcohol; (**d**), ML (low-dose MPs-A), 150 mL/kg BW MPs-A + 13 mL/kg BW alcohol; (**e**), MM (medium-dose MPs-A), 300 mL/kg BW MPs-A + 13 mL/kg BW alcohol; (**f**), MH (high-dose MPs-A), 600 mL/kg BW MPs-A + 13 mL/kg BW alcohol; (**g**), CL (low-dose CPs-D), 150 mL/kg BW CPs-D + 13 mL/kg BW alcohol; (**h**), CM (medium-dose CPs-D), 300 mL/kg BW CPs-D + 13 mL/kg BW alcohol; (**i**), CH (high-dose CPs-D), 600 mL/kg BW CPs-D + 13 mL/kg BW alcohol. black arrows and circles represent lipid droplets and inflammatory cell infiltration.

The hepatoprotective effect of MPs-A and CPs-D was further confirmed via histopathological examination. The liver histopathological sections (scale bar: 100 μm) stained with hematoxylin and eosin (H&E) (Figure 5A) indicated that the liver cells of the water control (WC) group were structurally intact, and the hepatic lobules were discernable. Nevertheless, hepatocellular swelling, loss of cell boundaries, fatty accumulation and explosive accumulation of inflammatory factors in the hepatic lobules were observed in the alcohol control (AC) group (fatty accumulation and lobular inflammation are marked with black arrows and circles, respectively). As expected, the administration of MPs-A and CPs-D showed effective protection against alcohol-induced liver injuries in a dose-dependent manner, which tended to ameliorate hepatic steatosis indicated by reducing hepatocyte edema, inflammatory cell infiltrates and the fat droplets in liver tissue. 

The results of liver histopathological sections (scale bar: 100 μm) stained with Oil Red O (ORO) (Figure 5B) were consistent with the results of hematoxylin and eosin (H&E) staining (lipid droplets are marked with black arrows). Numerous lipid droplets were observed in the alcohol control (AC) group and the administration of MPs-A and CPs-D reduced the lipid droplets in a dose-dependent manner. 

Based on the above observation, the high-dose MPs-A group (MH, 600 mg/kg BW MPs-A) was found to exhibit excellent hepatoprotective effects in the groups treated with different doses of MPs-A. Similarly, the high-dose CPs-D group (CH, 600 mg/kg BW CPs-D) was found to exhibit excellent hepatoprotective effects in the groups treated with different doses of CPs-D.

## 3. Discussion

Numerous pieces of evidence have shown that oxidative stress plays a major role in acute alcohol-induced liver injury by regulating lipid, protein, DNA and RNA levels and its effects on cellular dysfunction [34]. In other words, biological compounds with antioxidant activities provide a protective effect on the liver against free radical and alcohol-induced injuries. Therefore, the peptides from mussels (*Mytilus edulis*) (MPs) and clams (*Ruditapes philippinarum*) (CPs) hydrolyzed by five proteases (dispase, pepsin, trypsin, alcalase and papain) were prepared to evaluate their possible antioxidant and free radical scavenging activities in this study, which served as a crucial indicator of their underlying hepatoprotective activity. The results of hydroxyl radical (OH), 2,2-diphenyl-1-picrylhydrazyl (DPPH) and 2,2′-azino-bis(3-ethylbenzothiazoline-6-sulfonic acid) (ABTS) radical scavenging experiments clearly indicated that MPs and CPs exhibited excellent free radical scavenging activities in a dose-dependent manner. Similarly, many studies have also shown that the bioactive peptides of marine origin exhibit significant free radical scavenging activities [35,36,37,38,39,40]. For example, He et al. reported that the false abalone (*Volutharpa ampullacea perryi*) was hydrolyzed with different enzymes to extract antioxidant peptides. The results indicated that trypsin hydrolysates have the best biological activity and the strongest scavenging ability for ABTS radicals compared to pepsin, alcalase, neutrase and flavourzyme [38]. In addition, Upata et al. reported that enzymatic protein hydrolysate from jellyfish (*Lobonema smithii*) had high free radical scavenging activities, and the production of jellyfish hydrolysate using flavourzyme had the highest antioxidant activity [39]. 

According to the catalytic effect on the peptide chain, proteases are distinguished as endopeptidases and exopeptidases. Exopeptidases are so-called due to their site of action is only at the ends of peptide chains. In other words, they remove terminal amino acids only [40,41]. By contrast, endopeptidases cleave proteins at certain points along the chain and do not usually attack its end. Especially, alcalase and dispase are two typical endopeptidases that have been extensively used in the preparation of protein hydrolysates with strong antioxidant activities due to their broad specificity [25,42]. Indeed, the peptides hydrolyzed by alcalase and dispase exerted the strongest antioxidant activities in this study. Similarly, many studies have also shown that the bioactive peptides of marine origin hydrolyzed by alcalase and dispase exhibit superior antioxidant activities [9,43,44]. For example, Wang et al. reported that compared with the pepsin-hydrolyzed scallop (*Patinopecten yessoensis*) protein hydrolysates (SPH) and the dispase-hydrolyzed SPH, an Electron Spin Resonance (ESR) assay indicated that SPH hydrolyzed by alcalase had the best free radical scavenging effect based on a higher ratio of antioxidant amino acids (35.25%) and better solubility [43]. In addition, Wang et al. reported that peptides prepared from blue mussels (*Mytilus edulis*) were hydrolyzed by 4 varieties of proteases including alcalase, papain, pepsin and dispase, and the dispase-hydrolyzed peptides displayed the highest DPPH radical scavenging activity in comparison [9].

H_2_O_2_ can be transformed into hydroxyl radicals and oxygen free radicals, which have toxic effects on hepatocytes [45,46]. Therefore, the model of H_2_O_2_-induced oxidative damage in hepatocytes is often used for preliminary evaluation of hepatoprotective active substances. In this study, based on the above results of antioxidant activities of MPs and CPs, MPs hydrolyzed by alcalase (MPs-A) and CPs hydrolyzed by dispase (CPs-D) were chosen to evaluate their cytoprotective effects against oxidative damage in human hepatocellular carcinomas (HepG2) cells caused by H_2_O_2_. The result of the viability of HepG2 cells clearly indicated that cell damage induced by H_2_O_2_ was significantly alleviated by the addition of MPs-A and CPs-D, both peptides showed excellent in vitro hepatoprotective activity in the concentration range of 25–800 μg/mL. Similarly, many studies have also shown that the bioactive peptides of marine origin have a cytoprotective function on HepG2 cell damage [47,48,49]. For example, Xu et al. reported that hydrolysate was prepared from Asian clams (*Corbicula fluminea*) hydrolyzed by trypsin, and the peptide component separated from the low molecular weight part (<5 kDa) showed a significant protective effect on HepG2 cells with H_2_O_2_-induced oxidative damage. Such a positive effect was mainly attributed to the radical scavenging capability [50]. In addition, Hu et al. reported that the antioxidant peptides from grass carp (*Ctenopharyngodon idellus*) scale gelatin had a protective effect against the HepG2 cells’ oxidative damage induced by H_2_O_2_, which significantly promoted HepG2 cells growth and inhibited cell apoptosis [51].

In recent years, the model of alcohol-induced liver injury has been widely used to evaluate the hepatoprotective effect of active substances. It is widely considered that alcoholic liver injury is mediated by a variety of factors including accumulation of fat, oxidative damage, proinflammatory cytokines, increased collagen deposition and activation of various nonparenchymal cells [52]. Therefore, through the analysis of biochemical indicators in serum and liver, as well as the observation of hepatic histopathological sections, the hepatoprotective effects of MPs-A and CPs-D on acute alcohol-induced liver injury in mice were appraised in this study. This research confirmed that alcohol-fed mice (AC group) do not seem to gain as much weight as the control mice (WC group) in spite of alcohol feeding leading to increased liver index. Strikingly, MPs-A and CPs-D feeding effectively increase the gain in body weight resulting in a greater decrease in the liver index. Similarly, many studies have also shown that the bioactive peptides of marine origin have the effect of increasing the gain in body weight and reducing the liver index [53,54,55]. For example, Park et al. reported that compared with the body weight gain of the negative control mice treated with oral ethanol, the corresponding indicator was increased in the experimental mice treated with oral krill (*Euphausia superba*) protein hydrolysates. The result indicated that such hydrolysates may have a protective effect against alcohol-induced toxicity [54]. In addition, Gao et al. reported that the liver index of the negative control mice treated with oral alcohol was significantly higher than that of the water control group. In contrast, a high dose of peptides from oral oysters (*Crassostrea gigas*) hydrolyzed by alcalase could reduce the above-mentioned increase in liver index caused by alcohol exposure [55].

It has been widely accepted that the body protects itself from the oxidative stress induced by abundant drinking through enzymatic antioxidations, which are closely related to glutathione peroxidase (GSH-PX), superoxide dismutase (SOD), aspartate aminotransferase (AST) and alanine aminotransferase (ALT). GSH-PX exploits the thiol-reducing capacity of GSH to reduce oxidized lipids and proteins, thus contributing to H_2_O_2_ catabolism and detoxification of endogenous metabolic peroxides and hydroperoxides [56,57]. By contrast, SOD can inhibit the destruction of cell structure by free radicals because it can terminate the free radical chain reactions by scavenging superoxide radicals, hence it is one of the vital antioxidant enzymes in vivo [58]. As for ALT and AST, they can be released into the blood in large quantities and can be detected easily in serum when liver damage occurs, which are crucial indicators to evaluate the degree of liver injury [59]. Therefore, AST, ALT, GSH-PX and SOD activities are important indicators of oxidative stress in the progress of acute alcohol-induced liver injury [13,60]. In the present study, the serum ALT and AST activities were increased in the alcohol-intoxicated mice, and the supplementation with MPs-A and CPs-D could markedly reduce the serum ALT and AST levels caused by oral alcohol. Meanwhile, the hepatic SOD and GSH-PX activities were significantly reduced in the alcohol-intoxicated mice, and the treatment of MPs-A and CPs-D could effectively upregulate these two indicators. Similarly, many studies have also shown that the bioactive peptides of marine origin exerted the hepatoprotective effect by regulating the activities of the above-mentioned enzymes [61,62,63]. For example, Wang et al. reported that intragastric administration of alcohol could increase the activities of AST and ALT and decrease the activities of SOD and GSH-PX. Those changes were reversed by the co-administration of oyster (*Crassostrea talienwhanensis*) peptide (<3500 Da) [61]. In addition, Li et al. reported that tilapia (*Oreochromis* spp.) skin collagen polypeptide could effectively decrease the serum levels of ALT and AST caused by oral D-galactose, as well as increase the activities of hepatic SOD and GSH-PX [63].

Except for AST, ALT, GSH-PX and SOD, malondialdehyde (MDA) is also an important indicator of liver injury. MDA is a major reactive aldehyde resulting from the biofilm peroxidation process [59], which has been generally used as an indicator of tissue damage such as acute liver injury by a series of chain reactions [64]. In addition, liver injury can also contribute to intrahepatic diffusion of fatty acids, resulting in increased triacylglycerol (TG) and total cholesterol (TC) content in blood [31]. Obviously, the present study indicated that the treatment of MPs-A and CPs-D could effectively downregulate the serum levels of TG and TC and the hepatic MDA, which further confirmed that MPs-A and CPs-D could effectively inhibit fatty acid oxidation in the liver. Similarly, many studies have also shown that bioactive peptides of marine origin could inhibit intrahepatic diffusion of fatty acids and reduce the content of hepatic MDA [61,65]. For example, Lin et al. reported that peptides were prepared from salmon (*Oncorhynchus keta*) skin under the catalysis of complex protease (3000 U/g protein: 7% trypsin, 65% papain and 28% alkaline proteinase). Such peptides exhibited hepatoprotective effects on acute alcohol-induced liver injury in mice, including reducing the levels of TC, TG and MDA in liver hepatic and serum [65]. In addition, Wang et al. reported that oyster (*Crassostrea talienwhanensis*) peptide (<3500 Da) could significantly reduce the levels of MDA and TG compared to the alcohol-fed mice, further suggesting that the peptides had an effect on protecting the liver by inhibiting the oxidative stress and inflammatory response [61].

The results of serum and liver indicators measurements were also supported by histopathological observations. The result of the liver histopathological sections stained with hematoxylin and eosin (H&E) and Oil Red O (ORO) indicated that the liver tissues of alcohol-fed mice (AC group) exhibited severe pathological changes, such as extreme cellular swelling, loss of cell boundaries, inflammatory cell infiltration and fat droplet accumulation in the hepatic lobule, demonstrate that severe liver injury caused by heavy alcohol intake. The administration of MPs-A and CPs-D significantly alleviated the above phenomenon in a dose-dependent manner. Similarly, many studies have also shown that the bioactive peptides of marine origin could effectively alleviate the pathological and histological changes in the liver [55,66]. For example, Gao et al. reported that after oral administration of the peptides obtained from oyster (*Crassostrea gigas*) muscle hydrolyzed by alcalase to mice, the pathological changes (disordered liver cords, swollen hepatocytes, severe fat droplet accumulation and inflammatory cell infiltration) caused by alcohol could effectively be alleviated in a dose-dependent manner [55]. In addition, Bkhairia et al. reported the degenerative changes (sinusoidal congestion, hemorrhages, confluent necrosis and massive inflammatory cell infiltration around the perivenular area) involved in paracetamol-induced hepatic damage in rats. Fortunately, the peptides obtained from Golden grey mullet (*Liza aurata*) hydrolyzed by endogenous alkaline enzyme exhibited significant improvement of the above-mentioned changes [66].

Many conjectures have been proposed to explain the mechanisms of alcohol-induced hepatocyte injury, which is also helpful to clarify the hepatoprotective activities of various functional components. The most approbatory conjecture is the oxidative stress theory, which is involved in numerous diseases including alcoholic liver disease. During alcohol metabolism, reactive oxygen species, hydroxyethyl radicals and nitric oxide (NO) could contribute to oxidative stress associated with alcohol-induced liver injury [65,67]. Moreover, metabolizing alcohol in the liver would result in a series of abnormal physiological states, including an imbalanced state of redox, the oxidative stress state of the endoplasmic reticulum, and abnormal lipid metabolism of hepatocytes [58]. Consequently, swelling of hepatocytes, hepatic inflammation and fat droplet accumulation suggest hepatocyte injury by alcohol. Current experimental results demonstrated that compared with the corresponding indicators in negative control mice, higher contents of hepatic MDA, ALT and AST, as well as lower activities of hepatic SOD and GSH-PX were observed in experiment mice treated with MPs-A and CPs-D. This clearly indicated that anti-oxidative stress may involve in the hepatoprotective effect of MPs-A and CPs-D. Furthermore, serum TG and TC levels were gradually increased after oral administration of alcohol, which confirmed that excessive alcohol consumption could lead to a disturbance in lipid metabolism. Taken together, the results in this study indicated that by alleviating oxidative stress and lipid metabolism disturbance, MPs-A and CPs-D could effectively protect against acute alcoholic liver injury.

## 4. Materials and Methods

### 4.1. Materials and Chemicals

Mussels (*Mytilus edulis*) and clams (*Ruditapes philippinarum*) were purchased from Changxing market (Dalian, China). Dispase, pepsin, trypsin and alcalase were purchased from Beijing Solarbio Science & Technology Co., Ltd. (Beijing, China). Papain was purchased from Shanghai Aladdin Biochemical Technology Co., Ltd. (Shanghai, China). Dulbecco’s modified eagle medium (DMEM) cell culture medium was purchased from GIBCO Invitrogen Co., Ltd. (Carlsbad, CA, USA). Fetal bovine serum (FBS) was purchased from PAN-Biotech GmbH Co., Ltd. (Adenbach, Germany). Trypsin–EDTA (0.25%) and penicillin–streptomycin solution were purchased from Biological Industries Co., Ltd. (Kibbutz Beit Haemek, Israel). One × PBS (cell culture) and methyl thiazolyl tetrazolium (MTT) were purchased from Beijing Solarbio Science & Technology Co., Ltd. (Beijing, China). Dimethyl sulfoxide (DMSO) was purchased from Aladdin Reagent Co., Ltd. (Shanghai, China). H_2_O_2_ was purchased from Tianjin Damao Chemical Reagent Co., Ltd. (Tianjin, China). Glutathione (GSH) was purchased from Zhejiang Shenyou Biotechnology Co., Ltd. (Huzhou, China). Finally, 56 degrees of liquor was purchased from Beijing Hongxing Co., Ltd. (Beijing, China).

### 4.2. Assay Kits

Total cholesterol (TC), triacylglycerol (TG), aspartate aminotransferase (AST), alanine aminotransferase (ALT), malondialdehyde (MDA), glutathione peroxidase (GSH-PX) and superoxide dismutase (SOD) assay kits were purchased from the Nanjing Jiancheng Bioengineering Co., Ltd. (Nanjing, China).

### 4.3. Cells

HepG2 cells were purchased from Procell Life Science & Technology Co., Ltd. (Wuhan, China).

### 4.4. Animals

Seventy-two Kunming mice (18–22 g, male) were purchased from Liaoning Changsheng Biotechnology Co., Ltd. (Benxi, China). The animals were accommodated under standard environmental conditions (12 h:12 h L:D cycle at 25 ± 2 °C) with free access to standard food pellets and tap water ad libitum throughout the experimental period. All mice were acclimatized for a week prior to the experiments and were cared for and treated humanely. At the end of the experiment, the mice were sacrificed by CO_2_ asphyxiation in a covered container attached to a CO_2_ tank. In order to minimize the suffering and pain of the mice, the related experimental procedures were approved by the Animal Ethics Committee of Dalian Polytechnic University (DPU) and conducted in accordance with the Guidelines for Use and Care of Laboratory Animals of DPU.

### 4.5. Preparation of Peptides from Mussels (Mytilus edulis) and Clams (Ruditapes philippinarum) Hydrolyzed by Five Proteases

After separating into shells and meat by hand, the meats of mussels (*Mytilus edulis*) and clams (*Ruditapes philippinarum*) were crushed directly into minced meats. According to the result of our previous study [43], five kinds of proteases including pepsin, dispase, alcalase, trypsin and papain were selected as hydrolytic enzymes. Briefly, distilled water with pH adjusted (HCl or NaOH) for five proteases (pepsin—250 U/mg, pH 2.0; dispase—50 kU/g, pH 7.0; alcalase—200 kU/g, pH 8.0; trypsin—4 kU/g, pH 8.0 and papain—2 kU/mg, pH 6.0) were added to the minced meat in a ratio of 1:3 (meat/water, *w*/*v*, g/mL). Then, pepsin, dispase, alcalase, trypsin or papain was added to the system in a ratio of 1:0.06 (meat/protease, *w*/*w*, g/g). The hydrolysis reaction was carried out at optimum temperature (pepsin and trypsin—37 °C; dispase, alcalase and papain—50 °C) for 5 h. After 5 h, the reaction mixtures were placed in boiling water for 10 min, which was used to inactivate the proteases and also to terminate the enzymatic hydrolysis reaction. The obtained hydrolysates were cooled to room temperature and then centrifuged at 4 °C with 5000× *g* for 15 min. The supernatants were collected, which were subsequently placed in dialysis bags (intercept molecular weight of 5 kDa). Finally, the liquids in which the bags were immersed were lyophilized. Thus, the peptides from mussels (*Mytilus edulis*) (MPs) and clams (*Ruditapes philippinarum*) (CPs) hydrolyzed by five proteases were obtained, which were stored at −80 °C until further use.

### 4.6. Antioxidant Activities of MPs and CPs

MPs and CPs were dissolved in deionized water to prepare solutions with concentrations of 0.625, 1.250, 2.500, 5.000 and 10.000 mg/mL. Meanwhile, ascorbic acid was used as the positive control [43].

#### 4.6.1. Hydroxyl Radical (OH) Scavenging Activity

The hydroxyl radical (OH) scavenging activity assay used the method described by previous reports with slight modifications [68,69]. Briefly, 2 mL of MPs or CPs was mixed with 1, 10-phenanthroline (1.865 mM, 1 mL), FeSO_4_ (1.865 mM, 1 mL) and H_2_O_2_ (10 μM, 1 mL) and incubated at 37 °C for 1 h. Subsequently, the absorbance was read by using a microplate reader (infinite M200, TECAN, Switzerland) at 536 nm.

OH radical scavenging activity (%) = (As − A)/(Ac − A) × 100

where As represents the absorbance of samples, Ac represents the absorbance readings from the reaction system without H_2_O_2_ and A represents the absorbance readings from the reaction system without samples.

#### 4.6.2. DPPH Radical Scavenging Activity

The DPPH radical scavenging activity assay used the method described by previous reports with slight modifications [68,69]. Briefly, 0.5 mL of MPs or CPs was mixed with DPPH (200 μM, 0.5 mL) and incubated in the dark at room temperature for 30 min. Subsequently, the absorbance was read by using a microplate reader (infinite M200, TECAN, Switzerland) at 517 nm.

DPPH radical scavenging activity (%) = (A + Ae − As)/A × 100

where As represents the absorbance of samples, Ae represents the absorbance readings from samples substituted with 95% ethanol and A represents the absorbance readings from the reaction system without any sample.

#### 4.6.3. ABTS Radical Scavenging Activity

The ABTS radical scavenging activity assay used the method described by previous reports with slight modifications [68,69]. Briefly, the ABTS radical reagent solution was prepared at 7 mM with potassium persulphate (2.45 mM). The mixture was incubated in the dark and at room temperature for 16 h. The ABTS radical solution was diluted in 5 mM phosphate buffered saline (PBS) pH 7.4, to an absorbance of 0.70 ± 0.02 at 734 nm. Then, 0.5 mL MPs or CPs was adjusted using ABTS solution and incubated in the dark for 10 min. The absorbance was read by using a microplate reader (infinite M200, TECAN, Switzerland) at 734 nm.

ABTS radical scavenging activity (%) = (A − As)/A × 100

where As represents the absorbance of the reaction system with samples, A represents the absorbance of the reaction system without samples.

### 4.7. Hepatoprotective Effect of MPs and CPs on H_2_O_2_-Induced Oxidative Damage in HepG2 Cells

#### 4.7.1. Cell Culture and Treatments

The HepG2 cells were cultured in DMEM medium, supplemented with 10% (solution/solution, *v*/*v*, mL/mL) FBS and 1% (solution/solution, *v*/*v*, mL/mL) penicillin–streptomycin solution. The incubation conditions were shown as follows: pH—7.4, CO_2_—5%, temperature—37 °C and air atmosphere—95%. In particular, the medium was changed every 2–3 days. 

#### 4.7.2. Establishment of a H_2_O_2_-Induced HepG2 Cell Injury Model

H_2_O_2_-induced damage in the HepG2 cells model was established according to the method of Chen et al. and Ma et al. with slight modifications [32,70]. Briefly, the HepG2 cells were treated with different concentrations of H_2_O_2_ solutions (800, 1000, 1200, 1400 and 1600 μmol/L) for 4 h. Cell viability was determined using the MTT method to determine the appropriate H_2_O_2_ concentration (about 50% cell viability). Moreover, different concentrations of MPs or CPs (6 groups, 25, 50, 100, 200, 400 and 800 μg/mL) were also used to treat the HepG2 cells for 4 h (n = 8 for each group), which was used to evaluate whether these two hydrolysates have an adverse effect on HepG2 cells.

#### 4.7.3. Protective Effects of MPs and CPs on H_2_O_2_-Induced Oxidative Damage in HepG2 Cells

The HepG2 cells were divided into blank groups, control groups and experimental groups, which were as follows: blank group—HepG2 cells treated without H_2_O_2_ and peptides for 4 h; control group—HepG2 cells treated with H_2_O_2_ (1000 μmol/L) for 4 h; experimental group I—HepG2 cells treated with H_2_O_2_ (1000 μmol/L) and MPs (25, 50, 100, 200, 400 or 800 μg/mL) for 4 h; experimental group II—HepG2 cells treated with H_2_O_2_ (1000 μmol/L) and CPs (25, 50, 100, 200, 400 or 800 μg/mL) for 4 h. In particular, after performing filtering operations with 0.22 μm spin filters, H_2_O_2_ and MPs (or CPs) dissolved in DMEM medium were simultaneously added to the liquid mediums. The cell viabilities of all the above-mentioned samples were calculated, which was used to evaluate the effects of MPs and CPs on H_2_O_2_-induced oxidative damage in HepG2 cells. 

#### 4.7.4. Cell Viability Assay

After the HepG2 cells reached 70–90% confluence, they were harvested using trypsin and seeded into 96-well plates for 24 h at a density of 1 × 10^4^ cells/mL (100 μL per well). Subsequently, 50 µL of methyl thiazolyl tetrazolium (MTT) solution (5 mg/mL) was added to each well, and the plates were placed in a dark area to incubate at 37 °C. After 4 h, the supernatant was carefully discarded and 200 µL of dimethyl sulfoxide (DMSO) was added to each well. The plate was then shaken for 15 min at room temperature and the cell viability was estimated by using a microplate reader (infinite M200, TECAN, Switzerland) to read the absorbance at 490 nm.

Cell viability (%) = (As − A)/(Ac − A)

where As represents the absorbance value of sample-treated cells, Ac represents the absorbance value of non-treated cells, A represents the absorbance value with no cells.

### 4.8. Hepatoprotective Effects of MPs-A and CPs-D on Acute Alcohol-Induced Liver Injury in Mice

#### 4.8.1. Study Design

The mice were randomly divided into 9 groups with 8 mice in each group. The experiment period lasted for 10 days and was divided into two phases.

In the first phase (up to 7 days), the mice underwent daily intragastric administration with MPs-A and CPs-D. The doses for the 9 groups were as follows: WC (water control), isometric distilled water; AC (alcohol control), isometric distilled water; GC (GSH control), 150 mg/kg body weight (BW) GSH in distilled water; ML (low-dose MPs-A), 150 mg/kg BW MPs-A in distilled water; MM (medium-dose MPs-A), 300 mg/kg BW MPs-A in distilled water; MH (high-dose MPs-A), 600 mg/kg BW MPs-A in distilled water; CL (low-dose CPs-D), 150 mg/kg BW CPs-D in distilled water; CM (medium-dose CPs-D), 300 mg/kg BW CPs-D in distilled water; CH (high-dose CPs-D), 600 mg/kg BW CPs-D in distilled water. 

In the second phase (from 8 to 10 days), after 30 min of daily intragastric administration of water, MPs-A or CPs-D, aside from the WC group, which was given isometric distilled water, the groups were given 56 degrees of liquor daily (13 mL/kg BW). Body weight was measured at fixed times each day during the experiment.

Finally, retro-orbital blood samples were collected into tubes. After 2 h, all blood samples were centrifuged at 200× *g* for 15 min at 4 °C, and the resulting supernatants, designated serums, were carefully removed using a pipette. Subsequently, the mice were sacrificed by CO_2_ asphyxiation in a covered container that was attached to a CO_2_ tank. The livers were surgically removed and individually weighed. Furthermore, the liver index was calculated as the mass ratio of liver weight to body weight. Meanwhile, the above livers were used for histopathological analysis and preparation of 10% (solid/solution, *m*/*v*, g/mL) liver homogenate was prepared by homogenizing in 0.9% (solid/solution, *m*/*v*, g/mL) saline precooled in ice.

#### 4.8.2. Determination of Serum and Hepatic Biomarkers

Enzyme-linked immunosorbent assays (ELISAs) were used for the detection of serum biomarkers including total cholesterol (TC), triacylglycerol (TG), aspartate aminotransferase (AST) and alanine aminotransferase (ALT) as well as the hepatic biomarkers including malondialdehyde (MDA), glutathione peroxidase (GSH-PX) and superoxide dismutase (SOD). In addition, the protein content of liver tissues was measured by using a total protein (TP) quantitative assay kit. Follow the manufacturer’s instructions and read the relevant data on a microplate reader (infinite M200, TECAN, Switzerland). TC, TG, AST, ALT, MDA, GSH-PX and SOD were expressed as mmol/L, mmol/L, U/L, U/L, nmol/mg port, U/mg port and U/mg port, respectively. 

#### 4.8.3. Histopathologic Analysis

Liver tissues were fixed in 4% (solid/solution, *w*/*v*, g/mL) paraformaldehyde and embedded in paraffin wax. The paraffin sections (4 μm thick) were cut and each section was stained with the hematoxylin and eosin (H&E) technique. The other liver tissues were washed immediately with ice-cold PBS and embedded in the embedding compound at optimum cutting temperature (OCT). Liver tissues were cryosectioned (4 μm thick) and each section was stained with the Oil Red O (ORO) technique. The stained areas were observed with a microscope (Nikon Eclipse E100, Tokyo, Japan).

### 4.9. Statistical Analysis

The assay was carried out in triplicate. All data were expressed as the mean ± standard deviation. The results were statistically analyzed using one-way analysis of variance (ANOVA) followed by Dunnett’s test, with *p* < 0.05 considered significant. All statistics were performed using IBM SPSS Statistics version 26 (IBM Corp., Armonk, NY, USA).

## 5. Conclusions

In conclusion, the peptides from mussels (*Mytilus edulis*) (MPs) hydrolyzed by alcalase (MPs-A) and the peptides from clams (*Ruditapes philippinarum*) (CPs) hydrolyzed by dispase (CPs-D) had the highest antioxidant activities. Furthermore, the MPs-A and the CPs-D exhibited hepatoprotective effects in the H_2_O_2_-induced cell injury model and the mice model of acute liver injury. The present results clearly indicated that *Mytilus edulis* and *Ruditapes philippinarum* are good sources of hepatoprotective peptides. This will effectively improve the economic value of low-cost economical marine bivalve shellfish.

## Figures and Tables

**Table 1:** Effects of peptides from mussels (*Mytilus edulis*) hydrolyzed by alcalase (MPs-A) and clams (*Ruditapes philippinarum*) hydrolyzed by dispase (CPs-D) on body weight gain at the 10th day, liver index and serum indexes.

Group	Body Weight Gain on the 10th Day (g)	Liver Index (mg/g)	Serum Index			
			ALT (U/L)	AST (U/L)	TC (mmol/L)	TG (mmol/L)
WC	8.82 ± 1.36 ^a^	42.40 ± 2.19 ^b^	23.26 ± 5.02 ^b^	23.88 ± 7.62 ^bc^	4.12 ± 0.55 ^c^	1.97 ± 0.41 ^ab^
AC	5.35 ± 0.75 ^bc^	49.67 ± 5.27 ^a^	37.27 ± 9.60 ^a^	34.55 ± 7.98 ^a^	6.00 ± 1.77 ^a^	2.25 ± 0.64 ^a^
GC	6.46 ± 1.72 ^bc^	44.60 ± 1.84 ^ab^	27.67 ± 7.26 ^ab^	17.91 ± 2.28 ^ab^	4.15 ± 0.73 ^bc^	1.85 ± 0.36 ^abc^
ML	7.01 ± 1.44 ^ab^	46.70 ± 4.77 ^ab^	25.10 ± 7.68 ^b^	20.76 ± 11.21 ^c^	5.44 ± 0.80 ^abc^	1.57 ± 0.29 ^bc^
MM	5.84 ± 3.25 ^bc^	43.40 ± 4.53 ^b^	23.52 ± 6.58 ^b^	22.52 ± 7.96 ^bc^	4.77 ± 1.16 ^abc^	1.47 ± 0.31 ^bc^
MH	5.67 ± 2.43 ^bc^	49.75 ± 3.69 ^a^	23.82 ± 6.59 ^b^	18.80 ± 4.10 ^bc^	4.20 ± 0.49 ^bc^	1.37 ± 0.20 ^c^
CL	4.72 ± 0.91 ^c^	42.24 ± 8.33 ^b^	26.85 ± 9.76 ^ab^	25.23 ± 9.87 ^bc^	5.67 ± 1.93 ^ab^	1.79 ± 0.62 ^abc^
CM	4.50 ± 1.47 ^c^	45.35 ± 2.88 ^ab^	25.06 ± 8.69 ^b^	22.00 ± 5.75 ^bc^	5.64 ± 1.64 ^abc^	1.68 ± 0.41 ^bc^
CH	4.54 ± 1.93 ^c^	45.91 ± 1.46 ^ab^	23.84 ± 5.86 ^b^	20.33 ± 2.94 ^bc^	5.00 ± 1.28 ^abc^	1.69 ± 0.90 ^bc^

Note: WC (water control), isometric distilled water; AC (alcohol control), 13 mL/kg body weight (BW) alcohol; GC (GSH control), 150 mL/kg BW GSH + 13 mL/kg BW alcohol; ML (low-dose MPs-A), 150 mL/kg BW MPs-A + 13 mL/kg BW alcohol; MM (medium-dose MPs-A), 300 mL/kg BW MPs-A + 13 mL/kg BW alcohol; MH (high-dose MPs-A), 600 mL/kg BW MPs-A + 13 mL/kg BW alcohol; CL (low-dose CPs-D), 150 mL/kg BW CPs-D + 13 mL/kg BW alcohol; CM (medium-dose CPs-D), 300 mL/kg BW CPs-D + 13 mL/kg BW alcohol; CH (high-dose CPs-D), 600 mL/kg BW CPs-D + 13 mL/kg BW alcohol. Values of different groups with different letters in the same column are significantly different at *p* < 0.05.

## Data Availability

Research data are not shared.
